# *bub1* as a potential oncogene and a prognostic biomarker for neuroblastoma

**DOI:** 10.3389/fonc.2022.988415

**Published:** 2022-09-27

**Authors:** Jingjing Song, Chao Ni, Xubin Dong, Chenang Sheng, Yue Qu, Libin Zhu

**Affiliations:** ^1^ Department of Pediatric Surgery, the Second Affiliated Hospital and Yuying Children’s Hospital of Wenzhou Medical University, Wenzhou, China; ^2^ Department of Pediatric Allergy and Immunology, the Second Affiliated Hospital and Yuying Children’s Hospital of Wenzhou Medical University, Wenzhou, China; ^3^ Second Clinical College, Wenzhou Medical University, Wenzhou, China; ^4^ Department of Breast Surgery, the First Affiliated Hospital of Wenzhou Medical University, Wenzhou, China; ^5^ Wenzhou Medical University-Monash Biomedicine Discovery Institute (BDI) Alliance in Clinical and Experimental Biomedicine, Wenzhou, China

**Keywords:** *bub1*, neuroblastoma, oncogene, immunity, prognosis, biomarker

## Abstract

**Background:**

Neuroblastoma is the most common malignant extracranial tumor for children. Molecular mechanisms underpinning the pathogenesis of this disease are yet to be fully clarified. This study aimed to identify a novel oncogene that could be used as a biomarker informing the prognosis of neuroblastoma, and to predict its biological functions, using bioinformatics and molecular biology tools.

**Methods:**

Three data sets from the TARGET, GSE62564, and GSE85047 databases were used for analysis. Survivals of patients with high or low expression of *bub1* were compared, using the Kaplan-Meier curve and log-rank test. Immune infiltration was evaluated using ESTIMATE and MCP-counter algorithms. Synthetic small interfering RNAs (siRNAs) were employed to silence *bub1* expression in neuroblastoma cell lines SH-SY5Y and SK-N-SH, in order to characterize its biological functions. Gene enrichment analyses of *bub1* were carried out, using Gene Ontology (GO) and Kyoto Encyclopedia of Genes and Genomes (KEGG) analyses.

**Results:**

Expression of *bub1* was found to significantly affect overall survival and event-free survival of patients with neuroblastoma, positively correlate with the expressions of *tpx2* and the *ASPM* gene, and negatively correlate with host immune infiltration. Expression of *bub1* was elevated in patients with neuroblastoma. Silencing *bub1* expression using siRNAs in SH-SY5Y and SK-N-SH resulted in decreased cell growth (*p* < 0.05), reduced migration (*p* < 0.05), and increased apoptosis (*p* < 0.05). Function analysis of *bub1* revealed cancer-promoting effects, probably *via* regulating several important downstream molecules, including that related to the apoptosis process and epithelial-mesenchymal transition.

**Conclusion:**

We identified a potential tumor-promoting gene *bub1* for neuroblastoma that could also serve as a prognostic biomarker.

## Introduction

Neuroblastoma is the most common malignant extracranial solid tumor in children, occurring in eight per million children in the United States (1, 2). The most significant clinical feature of neuroblastoma is its diversity, with some tumors regressing from the primary tumor to mature and benign ganglioma and the others developing into a metastatic lethal disease (3). Whereas some patients have favorable prognoses (>90% survival, low to intermediate-risk), approximately 60% of all patients may develop high-risk diseases and have unfavorable 5-year survivals (4). Recurrence and cancer-related death were the most common outcomes of severe neuroblastoma (3). The survival rates of neuroblastoma were reported to be 81% for pediatric patients of younger ages (birth to 14 years old) and 57% for older patients (≥15 years old) (5). Higher survival rates were also found for patients who were diagnosed with early-stage neuroblastoma, except for newborns (6, 7). The clinical severity of neuroblastoma was reported to be related to tumor-related factors including cancer histology, cancer stage, classification, and cytogenetic characteristics (8). Several molecular and cytogenetic factors related to the prognosis of neuroblastoma have been proposed, including MYCN amplification, DNA content (ploidy), and changes in chromosome structure (9–11).

Immunotherapies such as chimeric monoclonal antibodies and chimeric antigen receptor (CAR) T cell therapy are tailored treatments for neuroblastoma, often showing less toxicity and higher efficacy in comparison to conventional therapies (12). Dinutuximab is an anti-disialylganglioside (GD2) chimeric monoclonal antibody that has been approved by the FDA for patients with high-risk neuroblastoma (13). Combinational usage of Dinutuximab and interleukin 2 (IL-2), granulosa cell macrophage colony-stimulating factor (GM-CSF), and isotretinoin have been found to significantly increase the survival of patients diagnosed with high-risk neuroblastoma (14, 15). Despite the unprecedented development of immunotherapies for neuroblastoma in the past decades, poor outcomes for neuroblastoma patients still remain a major concern (16, 17). This is partially due to the lack of accurate prognostic biomarkers that can guide the treatment for a variety of neuroblastoma cases.

Molecular biomarkers for tumors may allow early detection, diagnosis, and intervention of the disease, and subsequentially lead to a better treatment outcome (18, 19). Several molecular biomarkers associated with the occurrence and progression of neuroblastoma have been reported, including MYCN, a clinically recognized oncogenic transcription factor-related biomarker, anaplastic lymphoma kinase (ALK) (20), and PHOX2B (21, 22). MYCN has been used as a prognostic biomarker that predicts the effectiveness of anti-cancer immunotherapies, due to its association with host immune responses (23, 24). A considerable number of neuroblastoma patients, however, did not possess any of the above-mentioned biomarkers (23, 24); other biomarkers may exist in this population. The MYCN proto-oncogene regulates the expression of several vital genes involved in cell proliferation, including a circRNA’s target gene *bub1* (budding uninhibited by benzimidazoles 1) (25). Mutation of *bub1* has been linked to DNA aneuploidy, and alternative splicing resulted in multiple transcriptional variants (26–29). *bub1* has been proposed as a prognostic biomarker for other cancers including non-small cell lung cancer, gastric cancer, pancreatic cancer, and adrenocortical cancer (30–33). The expression of *bub1* was found to be able to reduce the immunosuppressive effect of these cancers (30–33). *bub1* expression was increased in human bladder cancer (BCa), and *bub1* kinase drives the progression and proliferation of BCa by regulating the transcriptional activation of STAT3 signaling (34). Owing to it association with MYCN, it is reasonable to speculate that *bub1* is also involved in the development of neuroblastoma and may be used to guide targeted immunotherapy against neuroblastoma.

This study aimed to clarify the role of *bub1* in the pathogenicity of neuroblastoma and as a prognostic biomarker for neuroblastoma.

## Materials and methods

### Data acquisition

Three public database cohorts were chosen for this study. The RNA sequence and clinical profile of neuroblastoma patients were obtained from the TARGET Children’s Tumor Database (https://ocg.cancer.gov/programs/target) (35). Two sequencing data sets (GSE62564 and GSE GSE85047) of neuroblastoma patients were from the GEO public functional genomics database (https://www.ncbi.nlm.nih.gov/geo/). The TARGET, GSE62564, and GSE85047 datasets included 249, 498, and 283 neuroblastoma tumor samples, respectively. Tumor staging was referred to the International Neuroblastoma Staging System (INSS) classification standard. The obtained clinical and pathological data were tabulated as follows: gender, age at diagnosis, INSS grade, MYCN expansion status, multiples in cells (ploidy), neuroblastoma risk classification [Children’s Oncology Group (COG) risk groups], histological prognosis, and Mitosis-Karyorrhexis Index (MKI).

### Cell culture and RNA interference

The neuroblastoma cell lines SH-SY5Y and SK-N-SH were purchased from Wuhan Procell Life Technology Co., Ltd. SH-SY5Y was cultured in a growth medium containing MEM/F12, 15% FBS, and 1% Penicillin/Streptomycin. A different growth medium with MEM, 10% FBS, and 1% Penicillin/Streptomycin was used for SK-N-SH. Both cell lines were incubated at 37°C in a 5% CO2 incubator and the growth medium was replenished every 48-72 h. Lipofectamine RNAiMAX transfection reagent (Thermo Fisher Scientific, USA) was mixed with siRNA to transfect neuroblastoma cells. The sequences of the si*-bub1* were as follows:

si*bub*1-1 (5’- GGGACUGUUGAUGCUCCAATT -3’, 5’- UUGGAGCAUCAACAGUCCCTT -3’), and

si*bub*1-2: (5’- GCAACAAACCAUGGAACUATT -3’, 5’- UAGUUCCAUGGUUUGUUGCTT -3’).

### RNA extraction and RT−qPCR

The total RNA of the cells was extracted using TRIzol reagent (Thermo Fisher Scientific, USA), per the manufacturer’s instructions. Isolated RNA samples were qualitatively and quantitatively assessed using the NanoDrop Microvolume Spectrophotometers (Thermo Fisher Scientific, USA) at 260/280 nm. The reverse transcription reaction was performed using ReverTra Ace qPCR RT Kit (Toyobo, Japan). Real-time qPCR (RT-qPCR) was carried out using 2×SYBR Green qPCR Mix (With ROX) (Sparkjade, China) and a fluorescence quantitative PCR instrument (Bio-rad, USA). The relative expression of the *bub1* mRNA was calculated using the 2-*ΔΔ*CT method and GAPDH as an endogenous control. The primers used for RT-qPCR were synthesized by Sangon Biotech (Shanghai) and listed below:

*bub1* forward primer, 5’-GAAAGCATGAGCAATGGGTAAA-3’;

*bub1* reverse primer, 5’- CCACCTGATGCAACTTCTTATG-3’;

*GAPDH* forward primer, 5’-GTCTCCTCTGACTTCAACAGCG-3’;

*GAPDH* reverse primer, 5’-ACCACCCTGTTGCTGTAGCCAA-3’.

### Western blot

RIPA buffer, protease inhibitors cocktail, and PMSF were all purchased from Beyotime Biotechnology, Shanghai, China. Cell lysates were centrifuged at 12,000 × g at 4 °C. Protein concentration was determined using the BCA protein assay. Proteins in the lysate were separated with a PAGE Gel Fast Preparation Kit (Epizyme, Shanghai, China) and subsequently electrotransferred to a PVDF membrane (Millipore, Bedford, MA). The PVDF membrane was blocked with 5% bovine serum albumin (Solarbio Science & Technology, Beijing, China). The target proteins were detected by incubating the membrane at 4°C overnight with primary anti-Bub1 antibody (at a dilution of 1:1000, Zen Bioscience), Bax antibody (at a dilution of 1:1000, Zen Bioscience), Bcl-2 (at a dilution of 1:1000, Affinity), E-cadherin (at a dilution of 1:1000, Proteintech), Vimentin (at a dilution of 1:1000, Proteintech), Phosphorylated GSK beta (at a dilution of 1:1000, Zen Bioscience), GSK3 beta (at a dilution of 1:2000, Zen Bioscience) and primary anti-GAPDH antibody (at a dilution of 1:5000, Bioworld Technology). A secondary antibody goat anti-rabbit IgG (at a dilution of 1:5000, Biosharp) or goat anti-mouse IgG (at a dilution of 1:5000, Solarbio) was added to the membrane and further incubated for two hours at room temperature. The protein bands were visualized using an ECL detection kit (Beyotime Biotechnology, Shanghai, China). The bands were scanned and photographed using the multifunctional gel imaging system (Bio-rad, USA) and quantitated with the Image Lab software (Bio-Rad).

### Immune cell infiltration

ESTIMATE algorithm was used to quantify the ratio of immunity to the matrix in the neuroblastoma microenvironment, following a published method (36). The TARGET neuroblastoma transcriptome profile was used to study differences in immune cell composition based on *bub1* expression. The MCP-counter algorithm was used to calculate the immune cell fraction of each sample, which reliably estimated the level of immune penetration. These algorithms were used to compare the level of immune cell infiltration between the *bub1* high and low expression groups. *p* < 0.05 was set as the criterion for selecting cases for subsequent analyses. The “ggpubr” R software package was used to analyze the immune infiltration scores between different *bub1* expression groups.

### Cellular proliferation, migration, and invasion assay

The CCK-8 assay (Shanghai Beyotime Biotechnology) was employed to assess cell viability. Transfected neuroblastoma cells (3 × 10^3^ cells per well) were seeded into 96-well microplates. The optical density (OD) was examined at 450 nm using an automatic enzyme label analyzer (Thermo Fisher Scientific, USA). For the colony formation assay, transfected neuroblastoma cells (3 × 10^3^ cells per well) were seeded into a 6-well plate and then incubated for 7-12 days till the cells grew into colonies. The plates were gently washed twice with phosphate-buffered saline (PBS), fixed with paraformaldehyde fixative solution for 20 minutes, stained with 0.1% crystal violet solution for 15 minutes, photographed, and counted using ImageJ software. Transwell plates (Corning, USA) were used for the migration assay. Cells (5 × 10^4^ cells per chamber) were seeded onto the upper chamber, and the growth medium with supplemented 10% FBS was added to the bottom chamber. After incubation for 48 hours at 37°C, non-migrated cells in the upper chamber were cautiously removed using a cotton swab. Migrated cells were then fixed with paraformaldehyde fixative solution for 20 minutes and stained with 0.1% crystal violet for 15 minutes. Migrated cells were examined with a microscope (40 x) at least 5 randomly selected vision fields for each well, photographed, and counted using ImageJ software.

### Apoptosis and flow cytometry assay

The neuroblastoma cell lines were collected after being transfected for 48 hours. Collected cells were washed with PBS three times, and resuspended with 300 μL of 1× binding buffer. Annexin V-fluorescein isothiocyanate (5 μl) and propidium iodide (5 μl) (BD Biosciences, USA) were added into the cell suspensions prior to 15 minutes of incubation at room temperature in the dark. The apoptosis rate was analyzed by FlowJo (Tree Star, USA) and defined as the percentage of Q2 + Q3.

### Bioinformatics and data analysis

The bioinformatics analyses were performed using R software (version 3.5.3), and the rest of the data were analyzed using Graphpad Prism 8.2.0 statistical software. *p* < 0.05 was considered statistically significant. One-way analysis of variance (ANOVA) and Mann–Whitney test were used to compare the means of two groups or multiple groups, depending on data distribution. A Chi-square test was used to compare demographic/clinicopathological presentations between *bub1* high- and low-expression groups. Overall survival data and event-free survival data were adopted for survival analysis and prognostic assessment, using the “survival” and “survminer” R package and Kaplan-Meier curve and log-rank tests. The clinicopathological data and *bub1* expression data were used for univariate and multivariate Cox regression analysis. Spearman Rho correlations analysis was carried out for *bub1*-related gene analysis and gene enrichment analysis, using the “cor.test” function in the R software. Genes with a spearman correlation coefficient > 0.5 or < -0.5 were selected. Gene Ontology (GO) and Kyoto Encyclopedia of Genes and Genomes (KEGG) analyses were performed using the “enrichGo” and “enrichKEGG” functions in the “clusterprofiler” software package, respectively. The “ggplot2” and “pheatmap” packages are used for visualization.

## Results

### Demographic and clinicopathological characteristics of enrolled patients

A total of 249 neuroblastoma patients were selected from the TARGET database. Cancer staging was carried out for all enrolled patients using INSS standards. Among these patients, 57.43% were male and 42.57% were female, with a median age of 2.7 years old (**Table 1**). Twelve point one percent of these patients had stage one neuroblastoma, 0.4% had stage three neuroblastoma, and 86.8% had stage four cancer (**Table 1**). None of these patients had a history of other malignant tumors.

**Table 1:** Demographic/clinicopathological features of the TARGET dataset used for this study.

		TARGET	Percentage (%)
**Gender**	Male	143	57.43
	Female	106	42.57
**Age**	<18 months	36	14.46
	≥18 months	213	85.54
**INSS Stage**	1	30	12.05
	2	0	0
	3	1	0.40
	4	216	86.75
	NA	2	0.80
**MYCN status**	No amplification	175	70.28
	Amplification	68	27.31
	NA	6	2.41
**Ploidy**	=1	63	25.30
	>1	104	41.77
	NA	82	32.93
**COG risk**	Low and Intermediate	217	87.15
	High	30	12.05
	NA	2	0.80
**Histology**	Favorable	13	5.22
	Unfavorable	170	68.27
	NA	66	26.51
**MKI**	Low	69	27.71
	Intermediate	55	22.09
	High	52	20.88
	NA	73	29.32
**Total**		249	100

NA, not applicable.

### Expression of bub1 was significantly related to clinicopathological characteristics of neuroblastoma

A total of 127 neuroblastoma patients with known clinicopathological characteristics and survival data were selected from the TARGET database and categorized into either *bub1* high expression (n=63) or *bub1* low expression (n=64) groups, using the median value of *bub1* expression in all patients as the cutoff (**Table 2**). Chi-square analysis was carried out to compare the clinicopathological characteristics of these two groups. Expression of *bub1* were found to be significantly related to older age (≥ 18 months, *p* = 0.0007), higher INSS classification (grade 3-4, *p* < 0.0001), more MYCN amplification (*p* < 0.0001), higher GOC risk (*p* = 0.0007), worse histological grade (undifferentiated, *p* = 0.0049), and higher mitotic fragmentation index (MKI) (*p* < 0.0001) (see **Table 2**).

**Table 2:** Correlation between bub1 expression levels and demographic/clinicopathological factors of neuroblastoma.

Demographic/clinicopathological factors		Low *bub1* expression	High-bub1	χ2	*p*
**Gender**	Male	41	30	3.482	0.062
	Female	23	33		
**Age**	<18 months	22	6	11.41	0.0007
	≥18 months	42	57		
**INSS Stage**	1-2	23	4	16.6	< 0.0001
	3-4	41	59		
**MYCN status**	No amplification	56	33	18.67	< 0.0001
	Amplification	8	30		
**Ploidy**	=1	17	25	2.469	0.1161
	>1	47	38		
**COG risk**	Low and Intermediate	41	59	16.6	< 0.0001
	High	23	4		
**Histology**	Favorable	10	1	7.908	0.0049
	Unfavorable	54	62		
**MKI**	Low	36	11	21.29	< 0.0001
	Median	16	24		
	High	12	28		
**Total**		64	63		

### bub1 as a prognostic factor for neuroblastoma

**Figure 1:**
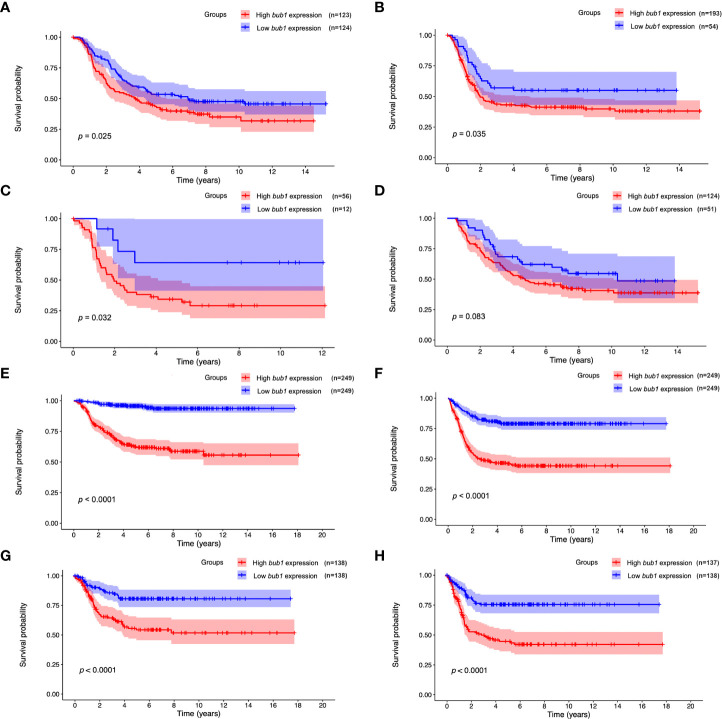
Prognostic value of *bub1* in neuroblastoma. **(A, B)** Kaplan–Meier curve using the TARGET dataset showed lower overall survival **(A)** and recurrence-free survival **(B)** in patients with highly expressed *bub1* relative to that with low *bub1* expression. **(C, D)** Kaplan-Meier curve using the TARGET dataset showed lower overall survival in high-*bub1*-expression patients with **(C)** or without **(D)** MYCN amplification in comparison with their low-*bub1*-expression counterparts. **(E, F)** Kaplan-Meier curve using the GSE62564 dataset showed lower overall survival **(E)** and recurrence-free survival **(F)** in patients with highly expressed *bub1* relative to that with low *bub1* expression. **(G, H)** Kaplan–Meier curve using the GSE85047 dataset showed lower overall survival **(G)** and recurrence-free survival **(H)** in patients with highly expressed *bub1* relative to that with low *bub1* expression.

Survival analysis was carried out for patients with high or low *bub1* expression respectively, using data from the TARGET (**Figures 1A-D**), GSE62564 (**Figures 1E, F**), and GSE85047 (**Figures 1G, H**) data sets. Results from three data sets all suggested that patients with high *bub1* expression had lower overall survival rates (**Figures 1A, E, G**) and recurrence-free survival rates (**Figures 1B, F, H**) than those with low *bub1* expression, implicating a potential role of *bub1* as a prognostic biomarker for patients with neuroblastoma.

Univariate Cox regression analysis found that the expression of *bub1*, MYCN amplification, ploidy (multiples in cells) > 1, and higher MKI were significant risk factors for poor overall survival (**Table 3**). Multivariate Cox regression analysis was not pursued as excessive hazard ratio (HR) values of the INSS classification and COG risk might have compromised the reliability of this test for other possible risk factors. To further clarify whether *bub1* expression was simply a coeffect of other risk factors such as MYCN amplification in predicting neuroblastoma survival, we divided patients into groups with or without MYCN amplification. In both groups, patients with high *bub1* expression showed lower overall survival rates than those with low *bub1* expression (**Figure 1C**, *p* = 0.032, and **Figure 1D**, *p* = 0.083), further supporting the role of *bub1* as an independent prognostic biomarker for neuroblastoma.

**Table 3:** Univariate Cox analysis of potential risk factors for poor survival of neuroblastoma patients.

	HR	95% CI	*p*
**Age (years)**	1.09	1.02-1.16	0.0075
**INSS Stage**	6694523.81	0-Inf	0.99
**Gender**	1.26	0.77-2.05	0.36
**Risk**	328130522	0-Inf	0.99
**MYCN status**	2.17	1.31-3.69	0.0025
**Ploidy**	0.42	0.25-0.68	0.00044
**Histology**	1.47	0.53-4.04	0.46
**MKI**	6	1.09-1.95	0.012
***bub1* expression**	1.53	1.13-2.07	0.0060

### bub1 expression and host immune infiltration in neuroblastoma

**Figure 2:**
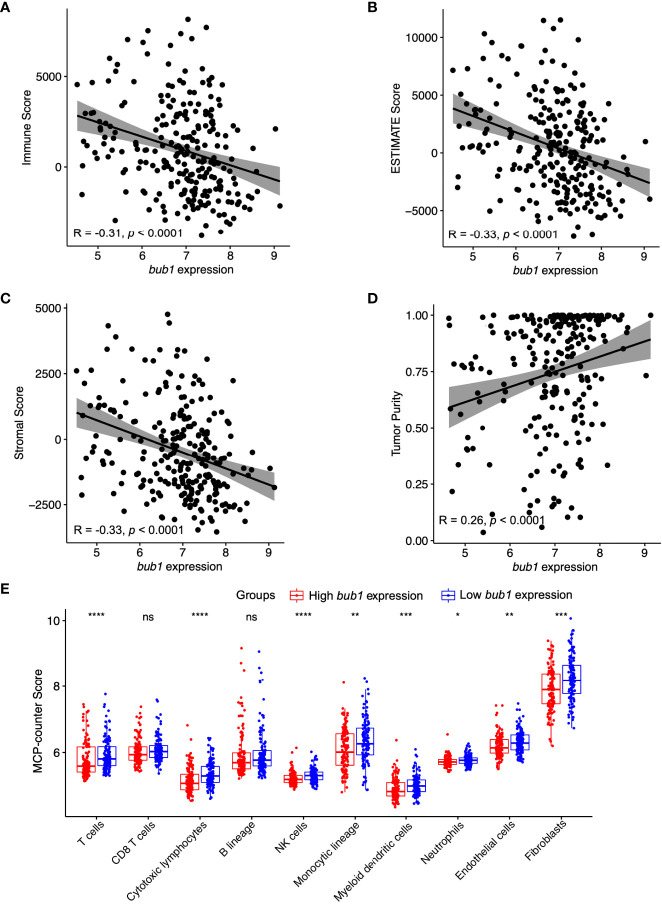
Correlation between *bub1* gene expression and immune infiltration. **(A-D)** Spearman’s correlation analysis revealed moderate negative correlations between *bub1* expression and the immune score **(A)**, ESTIMATE score **(B)**, and stromal score **(C)** respectively, and a weak positive correlation between *bub1* expression and the tumor purity **(D)**. **(E)** Profiles of tumor-infiltrating immune cells in neuroblastoma microenvironment assessed through MCP-counter algorithm *p ≤ 0.05, **p < 0.01, ***p < 0.001,****p < 0.0001, "ns" means “no sense”, p > 0.05.

The stromal score and immune score are two well-established parameters of host immune responses, referring to the relative percentage of stromal cells and immune cells in the tumor microenvironment (37). We used the ESTIMATE algorithm to calculate the stromal score and immune score in neuroblastoma samples. Spearman Rho correlations analyses found moderate negative correlations between *bub1* expression and the immune score, stromal score, and ESTIMATE score respectively (**Figures 2A-C**), and a weak positive correlation between *bub1* expression and the tumor purity (**Figure 2D**).

The MCP-counter algorithm was also used to calculate the immune cell score of neuroblastoma samples. Patients with low expression of *bub1* had higher counts of T cells, cytotoxic lymphocytes, monocyte lineage, natural killer cells, myeloid dendritic cells, endothelial cells, neutrophils, and fibroblasts than those with high expression of *bub1*, further supporting the negative correlation between immune cell scores and *bub1* expression (**Figure 2E**).

### Silencing bub1 inhibited the proliferation, migration, invasion, and apoptosis of neuroblastoma cells

**Figure 3:**
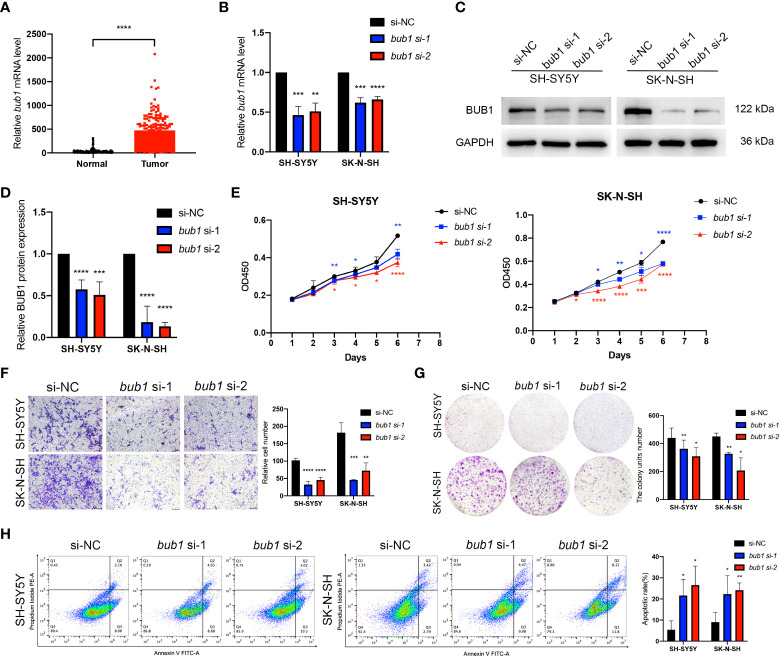
Expression of *bub1* promotes proliferation and migration of neuroblastoma cancer cells *in vitro*. Expression of *bub1* in the TARGET dataset **(A)**. RT-qPCR **(B)**, qualitative **(C)**, and quantitative **(D)** western blot analysis all suggested that siRNA successfully degraded *bub1* mRNA and prevented Bub1 protein synthesis in two cancer cell lines. NC, negative control. **(E)** CCK8 cell proliferation experiment after interfering *bub1* expression using siRNA in SH-SY5Y and SK-N-SH cells. **(F)** Qualitative analysis of cell migration after interfering *bub1* expression using siRNA in SH-SY5Y and SK-N-SH cells. Cells were stained with 0.1% crystal violet solution and examined with a light microscope. Magnification = 40 x. **(G)** Quantitative analysis of cell proliferation using colony formation assay showed significantly fewer colonies formed by *bub1*-silenced SH-SY5Y and SK-N-SH cells relative to their untreated controls. **(H)** Flow cytometry showed that *bub1* silencing increased early apoptosis of SH-SY5Y, and early and late apoptosis of SK-N-SH cells. Early and late apoptotic cells were characterized respectively as PI-/Annexin V+ and PI+/Annexin V+. All experiments were carried out in three biological repeats. Data are presented as the mean ± standard deviation (SD). *p < 0.05, **p < 0.01, ***p < 0.001, ****p < 0.0001.

Expression of *bub1* was elevated in patients with neuroblastoma in the TARGET dataset (**Figure 3A**). In order to verify the interference efficiency of *bub1* siRNA in neuroblastoma cells, we transfected two *bub1*-specific siRNA into SH-SY5Y and SK-N-SH cells respectively. RT-qPCR (**Figure 3B**, inhibition efficiency > 30%, *p* < 0.01) and western blot (**Figures 3C, D**) assays both showed significant inhibition of endogenous *bub1* expression in SH-SY5Y and SK-N-SH. CCK8 cell proliferation assay found that the silencing of *bub1* by siRNA significantly reduced the growth of SH-SY5Y and SK-N-SH, with the most evident effect observed on day 6 (**Figure 3E**). To further explore the impact of *bub1* silencing on metastasis, a transwell assay was set up to evaluate the migration and invasiveness of cancer cells. *bub1* silencing significantly hindered the migration and invasiveness of SH-SY5Y and SK-N-SH (**Figure 3F**). Colony formation assays further suggested reduced proliferation of si-*bub1* neuroblastoma cells relative to its si-NC counterparts (**Figure 3G**). Flow cytometry was carried out to compare cell apoptosis in the *bub1* silenced group and the control group. The proportion of cells undergoing apoptosis was low (<10%) for both groups. *bub1* silencing enhanced early apoptosis of SH-SY5Y cells, and early and late apoptosis for SK-N-SH cells (**Figure 3H**).

### Gene enrichment analysis of bub1

**Figure 4:**
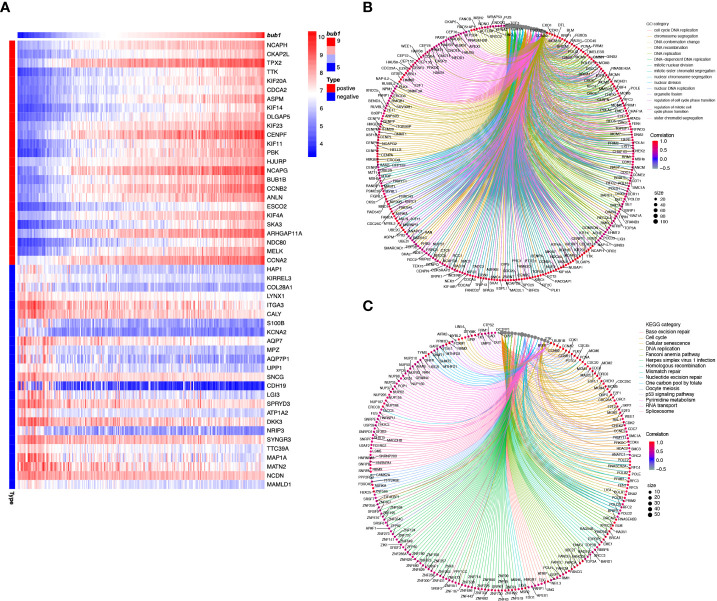
Molecular function analysis of *bub1* in neuroblastoma. **(A)** Enrichment heat map of *bub1*-related genes. Genes strongly co-expressed with *bub1* are exhibited in order of Spearman’s correlation coefficient. **(B)** Gene ontology (Go) enrichment analysis of *bub1*. **(C)** Kyoto Encyclopedia of Genes and Genomes (KEGG) analysis of *bub1*.

In order to clarify the molecular functions of *bub1* in neuroblastoma, we used the TARGET data set and searched for genes that were closely related to *bub1*. A total of 975 genes were obtained from co-expression analysis. Using gene enrichment analysis, the top 25 genes most relevant to *bub1* in neuroblastoma were presented as heat maps (**Figure 4A**). *bub1* was found to be positively correlated with genes encoding signal molecules vital for mitosis and chromosome assembly, such as NCAPH, CKAP2L, TPX2, TTK, KIF20A, CDCA2, ASPM, KIF14, and DLGAP5. *bub1* was also found to be negatively correlated with signal molecules important for neurotransmitter and signal transmission such as HAP1, KIRREL3, COL28A1, LYNX1, ITGA3, CALY, S100B, KCNA2, AQP7, and MPZ.

We selected genes that had a strong correlation with *bub1* (Spearman correlation > 0.5, *p* < 0.001) for further GO and KEGG analyses (**Figures 4B, C**). GO enrichment analysis showed that *bub1* was primarily involved in DNA replication, chromosome segregation, nuclear division, DNA-dependent DNA replication, organelle fission, mitotic nuclear division, nuclear chromosome segregation, DNA conformation changes, sister chromatid segregation, cell cycle DNA replication, and other biological processes (BP). Bub1 was found to be the cellular component of the chromosomal region, chromosome, centromeric region, condensed chromosome, spindle, kinetochore, concentrated chromosome kinetochore, replication fork, spindle pole, mitotic spindle, microtubule, telomeric region, and so on. The molecular function (MF) enriched by Bub1 included the catalytic activity, acting on DNA, DNA-dependent ATPase activity, ATPase activity, DNA helicase activity, helicase activity, single-stranded DNA binding, DNA replication origin binding, single-stranded DNA helicase activity, tubulin binding, DNA secondary structure binding, damaged DNA binding, histone binding, microtubule-binding, etc. (**Figure 4B**; **Supplementary Table 1**). KEGG enrichment analysis showed that *bub1* was mainly involved in base excision repair, cell cycle, cell senescence, DNA replication, Fanconi anemia pathway, herpes simplex virus 1 infection, homologous recombination, mismatch repair, nucleotide excision repair, one carbon pool by folate, oocyte meiosis, p53 signaling pathway, pyrimidine metabolism, RNA transport and spliceosome processes (**Figure 4C**; **Supplementary Table 2**).

### bub1 silencing affected epithelial-mesenchymal transition, cell apoptosis, and the Wnt signaling pathway

**Figure 5:**
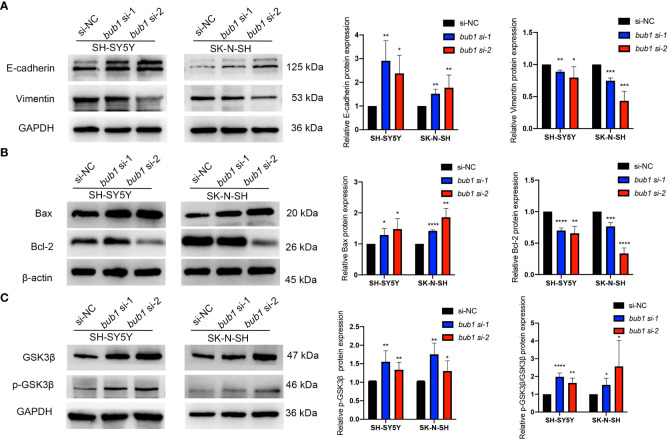
Bub1 participates in EMT, apoptosis, and other pathways. **(A)** Expression of EMT-related proteins E-cadherin and vimentin after silencing *bub1* in two cell lines. **(B)** Expression of apoptosis and proliferation-related proteins Bax and Bcl-2 after silencing *bub1* in two cell lines. **(C)** Expression of GSK3β and p-GSK3β proteins after silencing *bub1* in two cell lines. Representative images of Western blotting are shown in the left panels. Quantitative analyses of Western blotting are displayed in the right panels. All western blotting experiments were carried out in at least three biological repeats. Data are presented as the mean ± SD. *p < 0.05, **p < 0.01, ***p < 0.001, ****p < 0.0001.

The expression of *bub1* significantly affected the expression of proteins involved in epithelial-mesenchymal transition (EMT), cell apoptosis, and the Wnt signaling pathway, three biological underpinnings of the pathogenesis of neuroblastoma. Western blot showed decreased expression of mesenchymal marker vimentin and apoptosis-inhibiting Bcl-2, and increased expression of epithelial marker E-cadherin, apoptotic activators Bax and GSK3β, p-GSK3β and p-GSK3β/GSK3β in SH-SY5Y and SK-N-SH cells whose *bub1* expression has been silenced (**Figure 5**).

## Discussion

Neuroblastoma is a neuroendocrine tumor with varying severities and biological characteristics (38). Despite significant advances in surgical treatment and immunotherapies and the wide clinical application of these interventions, cancer recurrence, metastasis, and cancer-related death are still frequently reported (13, 15, 39, 40). This study evaluated *bub1* as an oncogene and a prognostic biomarker for neuroblastoma. Key findings of this study include 1) expression of *bub1* was significantly related to the development and clinicopathological characteristics of neuroblastoma, 2) *bub1* demonstrated a prognostic value for patient survival, 3) expression of *bub1* was associated with host immune infiltration of neuroblastoma microenvironment, 4) *bub1* exerted its biological function possibly by regulating the expression of proteins related to EMT, cell apoptosis and the Wnt signaling pathway.

Using bioinformatics, molecular biological tools, and public datasets, we successfully identified a new oncogene *bub1* in neuroblastoma patients. High *bub1* expression was found to be associated with clinicopathological development of neuroblastoma in children. Pediatric patients with highly expressed *bub1* had lower overall survival and recurrence-free survival, in comparison with those with low *bub1* expression. Further regression analyses suggested that *bub1* expression was a risk factor irrelevant to MYCN amplification for the poor survival of neuroblastoma patients, supporting its role as an independent prognostic biomarker.

Several algorithms have been established and used by others to study host immune responses in the neuroblastoma microenvironment, including the ESTIMATE algorithm (36), MCP-counter algorithm, and TIMER and quanTIseq algorithms (41). We employed TARGET neuroblastoma RNA-Seq data to study the relationship between *bub1* expression and host immune infiltration. A negative correlation between *bub1* expression and immune infiltration of the cancer microenvironment was found. It has been reported that lymphocyte infiltration and cancer survival were positively correlated in pediatric patients (42, 43). High expression of *bub1* in neuroblastoma thus may predict poor prognosis of the patient.

It has been reported that Bub1 has a variety of unique molecular functions in the cell cycle, primarily in the spindle assembly checkpoints and metaphase chromosome alignment (44). Disturbed mitotic checkpoints are a common feature of many human cancers. Mutations in the spindle checkpoint have been found in > 90% of all solid tumors and often lead to chromosomal instability and aneuploidy (45–47). Others have reported that *bub1* might exert its biological functions by affecting chromosome instability and aneuploidy, apoptosis, and cell cycle signaling pathway (44). Knockout of *bub1* in p53-damaged cells such as HeLa cells led to aneuploidy (48). We carried out a gene enrichment analysis for *bub1*. GO and KEGG analyses suggested that *bub1* might participate in the p53 signaling pathway and chromosome segregation. We also identified several genes that were co-expressed with *bub1*. Among them were *tpx2* and *ASPM*. *tpx2* encodes a microtubule-associated protein that is often overexpressed in other cancers (49–52) and contributes to the growth, metastasis, recurrence, and poor prognosis of liver cancer (50, 53–55). Defect in *ASPM* was reported to be associated with autosomal-related recessive primary microcephaly (56, 57).

It was noticed that *bub1* affected the proliferation and migration of neuroblastoma cells. In order to gain more insight into the biological functions of *bub1*, we silenced *bub1* in two neuroblastoma cell lines SH-SY5Y and SK-N-SH. Inhibition of cell growth and weakened cell migration were noticed. Less cell growth was possibly due to stronger apoptosis of neuroblastoma cells induced by up-regulated expression of Bax protein and down-regulated expression of Bcl-2 protein. Both Bax and Bcl-2 belong to the Bcl-2 protein family and are key mediators of the apoptosis pathway (58, 59). The effect of *bub1* on weakened cell migration might be mediated by up-regulated expression of epithelial markers (E-cadherin) and down-regulated expression of the mesenchymal marker vimentin. Down-regulation of E-cadherin may lead to weaker cell adhesion to the tissue and greater cell motility, which allows cancer cells to penetrate the basement membrane and invade surrounding tissues (60).

Bub1 might be involved in some other signal pathways related to neuroma tumor cells derived from neural crest cells and mediated by GSK3, such as PI3K/Akt, Notch, mTOR, insulin, Wnt, Shh, receptor tyrosine kinase, mitogen-activated protein kinase, and p53 pathways (61, 62). GSK3 is a negative mediator of the Wnt signaling pathway that has known importance in the induction, stratification, and differentiation of neural crest cells (61, 63). GSK3 is also the main regulator of neural progenitor cell homeostasis, integrating a variety of proliferation and differentiation signals (64). Our study of pediatric neuroblastoma found that silencing *bub1* led to increased expression of GSK3β and p-GSK3β and suppressed expression of p-GSK3β/GSK3β. Similar effects of Bub1 have been reported for breast cancer (65) and Hela cells (66).

A major limitation of this study was that we used public datasets and did not include our own patient data for further verification. Another limitation was that no direct experimental evidence has been provided to support the role of *bub1* in neuroblastoma immune infiltration. In addition to apoptosis, EMT, and Wnt signaling pathway, other mechanisms driven by *bub1* expression and underpinning the development of neuroblastoma may also exist. Future studies are needed to address these limitations.

## Conclusion

Using bioinformatics and molecular biological tools, we identified *bub1*, a tumor-related gene in neuroblastoma. *bub1* may function as an oncogene by regulating the expression of important pathogenesis-related proteins, including those involved in EMT, cell apoptosis, and the Wnt signaling pathway. Our research also revealed that *bub1* may be used as a potential prognostic biomarker. A future in-depth study using our patient data will deepen our understanding of the role of *bub1* in the pathogenesis and prognosis of neuroblastoma.

## Data availability statement

Publicly available datasets were analyzed in this study. This data can be found here: TARGET, GSE62564, and GSE85047 databases.

## Author contributions

LZ designed the study. JS, CN, and XD obtained and analyzed the data. JS, CS, and YQ drafted the article. LZ, CN, and XD reviewed and revised the manuscript. All authors listed approved the submitted version.

## Funding

This work was supported by the Health Commission of Zhejiang Province (NO.2018KY128), the Wenzhou Science and Technology Bureau (NO.Y2020075), and the Zhejiang Province welfare technology applied research project (LGF21H040009).

## Acknowledgments

The authors would like to thank all team members of the TARGET-neuroblastoma, GSE62564, and GSE85047 projects for providing public-accessible data.

## Conflict of interest

The authors declare that the research was conducted in the absence of any commercial or financial relationships that could be construed as a potential conflict of interest.

## Publisher’s note

All claims expressed in this article are solely those of the authors and do not necessarily represent those of their affiliated organizations, or those of the publisher, the editors and the reviewers. Any product that may be evaluated in this article, or claim that may be made by its manufacturer, is not guaranteed or endorsed by the publisher.
